# A roadmap for gene system development in *Clostridium*

**DOI:** 10.1016/j.anaerobe.2016.05.011

**Published:** 2016-10

**Authors:** Nigel P. Minton, Muhammad Ehsaan, Christopher M. Humphreys, Gareth T. Little, Jonathan Baker, Anne M. Henstra, Fungmin Liew, Michelle L. Kelly, Lili Sheng, Katrin Schwarz, Ying Zhang

**Affiliations:** a Clostridia Research Group, BBSRC/EPSRC Synthetic Biology Research Centre, School of Life Sciences, University of Nottingham, Nottingham, NG7 2RD, UK; b Nottingham Digestive Disease Centre, NIHR Biomedical Research Unit, The University of Nottingham, University Park, Nottingham, UK

**Keywords:** Restriction modification, Gene transfer, ClosTron, Allelic exchange, Counterselection marker, *pyrE*, Knock-out, Knock-in, Fluoroorotic acid

## Abstract

*Clostridium* species are both heroes and villains. Some cause serious human and animal diseases, those present in the gut microbiota generally contribute to health and wellbeing, while others represent useful industrial chassis for the production of chemicals and fuels. To understand, counter or exploit, there is a fundamental requirement for effective systems that may be used for directed or random genome modifications. We have formulated a simple roadmap whereby the necessary gene systems maybe developed and deployed. At its heart is the use of ‘pseudo-suicide’ vectors and the creation of a *pyrE* mutant (a uracil auxotroph), initially aided by ClosTron technology, but ultimately made using a special form of allelic exchange termed ACE (Allele-Coupled Exchange). All mutants, regardless of the mutagen employed, are made in this host. This is because through the use of ACE vectors, mutants can be rapidly complemented concomitant with correction of the *pyrE* allele and restoration of uracil prototrophy. This avoids the phenotypic effects frequently observed with high copy number plasmids and dispenses with the need to add antibiotic to ensure plasmid retention. Once available, the *pyrE* host may be used to stably insert all manner of application specific modules. Examples include, a sigma factor to allow deployment of a *mariner* transposon, hydrolases involved in biomass deconstruction and therapeutic genes in cancer delivery vehicles. To date, provided DNA transfer is obtained, we have not encountered any clostridial species where this technology cannot be applied. These include, *Clostridium difficile, Clostridium acetobutylicum, Clostridium beijerinckii*, *Clostridium botulinum*, *Clostridium perfringens, Clostridium sporogenes, Clostridium pasteurianum*, *Clostridium ljungdahlii*, *Clostridium autoethanogenum* and even *Geobacillus thermoglucosidasius*.

## Highlights

• A simple roadmap for the development and deployment of gene systems in clostridia.
• Allelic exchange using *pyrE* alleles and pseudo-suicide vectors.
• Knock-out and knock-in using allele-coupled exchange (ACE).
• Complementation studies through genome insertion.
• Genome insertion of application specific modules.

## 1 Introduction

The genus *Clostridium* have long been recognised as a large and disparate grouping of bacteria that has representatives of importance to both human and animal diseases as well as to the industrial production of chemicals and fuels. Whilst the majority of those species responsible for human and animal diseases have been known for decades, in recent years the importance of members of the class clostridia in the gut microbiome has become ever more apparent [1], [2]. On the other hand, the desire to exploit an ever widening diversity of species for biotechnological purposes has intensified. Of particular note, is the growing momentum behind the industrialisation of gas fermentation for chemical and fuel production using clostridial acetogens. Acetogenic bacteria, typified by *Clostridium autoethanogenum* [3], are able to capture carbon (CO or CO_2_) through gas fermentation, allowing them to grow on a spectrum of waste gases from industry (*eg*., steel manufacture and oil refining, coal and natural gas) to produce ethanol [4], [5]. They can also consume ‘synthesis gas’ (CO and H_2_) made from the gasification of renewable/sustainable resources, such as biomass and domestic/agricultural waste. Acetogenic gas fermentation can, therefore, produce ethanol in any geographic region without competing for food or land. Indeed, the commercialisation of ethanol production from ArcelorMittal Steel Mill off-gas is now at an advanced stage [6]. When fully scaled, it could enable the production in Europe of around 500,000 tons of ethanol a year. Intriguingly, *Clostridium difficile* carries the pivotal genes required for CO/CO_2_ fixation, the Wood Ljungdahl pathway, and is reported to be able to grow on CO as a carbon source [7].

Given the increasing numbers of clostridial species that need to be more thoroughly characterised, either to counter the diseases they cause or to exploit their beneficial properties, the implementation of genetic systems is required. Accordingly, SBRC Nottingham has formulated a roadmap for gene system development in any clostridial species.

## 2 Roadmap basis

The roadmap revolves around exploitation of the dual, and opposite, phenotypes conferred on the cell by mutant and wildtype *pyrE* alleles. The presence of the former makes the host a uracil auxotroph that is resistant (^R^) to fluoroorotic acid (FOA), while the latter confers sensitivity (^S^) to FOA but is a uracil prototroph. Selective cycling between these two alleles allows recombination-based genome editing by ‘knock-out’ (KO) and ‘knock-in’ (KI). Importantly, the availability of a mutant *pyrE* allele may be used as a locus for the rapid genome insertion of DNA for both complementation studies and for the insertion of application specific modules. Using *pyrE* mutant hosts, therefore, presents considerable advantages for all mutational studies, regardless of the mutagen employed. Implementation of the roadmap is reliant on two fundamental developments being in place, namely: (i) the availability of a fully annotated genome sequence, and; (ii) a means of introducing DNA into the clostridial host.

### 2.1 Genome sequences

The availability of an annotated genome sequence is central to gene system development, not only to identify gene targets, but additional to assist in overcoming RM barriers (see 2.2). The closure of whole genome sequences, however, is hindered by the presence of long stretches of repetitive DNA which can prevent scaffold assembly of the shorter DNA reads generated by commonly used technologies, such as, Illumina MiSeq, Ion Torrent and 454 GS FLX + Titanium. In these cases, the read lengths generated (100–1000 base pairs) are unable to cover the repetitive sequence lengths of 5–7 Kb commonly found in bacteria [8]. Genome closure therefore requires expensive and time consuming manual finishing. PacBio have developed Single Molecule Sequencing Technology (SMRT) which is capable of generating read lengths in excess of 15 Kb [9] and currently, therefore, represents the technology of choice for determining whole genomes. However, compared to Illumina sequencing [10], [11] the error rate for PacBio sequencing is relatively high, particularly across homopolymer regions between two and fourteen base pairs in length [11]. Accordingly, it is advisable to combine PacBio sequencing with Illumina sequencing, mapping the latter reads to the PacBio-derived, reference assembly. Following the correction of any errors in the determined closed genome, it can be submitted to one of a number of online facilities for automated annotation. For example, the Integrated Microbial Genomes (IMG) system at DOE’s Joint Genome Institute (JGI) provides such an annotation service (https://img.jgi.doe.gov/cgi-bin/mer/main.cgi).

It should be noted, that in those instances where the genome sequence of the clostridial species being used is published, the assumption should not be made that the sequence is correct. For instance, the first clostridial genome sequence to be determined (that of *Clostridium acetobutylicum* ATCC 824 [12], was recently shown to contain numerous errors in the form of 175 Single Nucleotide Variations (SNVs) and 48 insertions/deletions (Indels). Similarly, the PacBio-derived genome sequence of *Clostridium autoethanogenum* DSM 10061 (NCBI: GCA_000484505.1) was shown to contain 243 SNVs [13]. It is equally important, not to make the assumption that the laboratory isolate being used has an identical sequence to that published. SNVs and Indels can arise, particularly if the strain has been passaged (intentionally or otherwise) through single colonies. Thus, a strain of *Clostridium acetobutylicum* ATCC 824 in common use in a number of European laboratories (ATCC 824 COSMIC) was shown to possess 2 SNVs and 1 deletion in comparison to the strain deposited at the ATCC [13]. More telling are the changes identified in the erythromycin resistance derivative, 630Δ*erm*, of the *Clostridium difficile* strain 630. These equated to 71 differences between the two strains, encompassing 8 deletions (including the Δ*erm* mutation), 10 insertions, 2 insertion-deletions, 50 substitutions and 1 region of complex structural variation [14].

### 2.2 Restriction/modification (RM) barriers

Prevention of DNA transfer by host RM systems is highly strain specific. Indeed, there are many instances where restriction has not been a problem, *e.g. Clostridium beijerinckii* NCIMB 8052 [15], *Clostridium perfringens* strain 13 [16], *C. difficile* strains CD37 [17] & CD630 [18] and *Clostridium botulinum* ATCC 3502 [19]. Genome sequencing has shown that many of these organisms carry at least one type II methylase gene (most often more than one), but they lack genes encoding the cognate restriction enzymes. Thus, for instance, the genomes of *C. botulinum* ATCC 3502 (NC_009495) and *C. perfringens* strain 13 (NC_003366) contain orphan copies of methylase genes (three and one, respectively), and are both readily transformable in the absence of any measures to circumvent restriction barriers [16], [20]. In many instances, however, the successful transfer of extrachromosomal elements, either by transformation or conjugation, has required the circumvention of the activity of endogenous restriction-modification (RM) systems. This is achieved through appropriate methylation of the vector DNA to be introduced. The nature and specificity of those enzymes involved has been achieved in a variety of ways. In early work, experimental approaches predominated in which restriction activity was initially detected in bacterial lysates after which, the restriction and the methylation specificity of the RM system was determined and then countered through the deployment of an appropriate methylase activity in the *Escherichia coli* donor with the requisite specificity. Early examples include *C. acetobutylicum* ATCC 824 [21], *Clostridium cellulolyticum* ATCC 35319 [22], *C. botulinum* ATCC 25765 [23] and *C. difficile* CD3 and CD6 [17] and more recently *Clostridium pasteurianum* [24] and *Clostridium cellulovorans* [25].

In recent years, there has been an increasing emphasis on using genome sequences to identify potential RM systems and using available gene knock-out systems to inactivate the identified restriction systems. Thus, successful DNA transfer in *C. cellulolyticum* was achieved by the inactivation of a putative MspI-like endonuclease gene, *ccel2866* [26], while DNA transfer in *C. acetobutylicum* ATCC 824 and DSM 1731 (essentially the same strain) in the absence of methylation of the transferred plasmid was achieved by inactivation of gene (CA_C1502) encoding type II restriction endonuclease Cac824I (recognition sequence GCNGC) using either ClosTron mutagenesis [27] or allelic exchange [28]. In the latter study, inactivation of a second gene encoding an additional type II restriction enzyme (Cac824II – recognition sequence CTGAAG) led to a further 8-fold increase in electroporation frequency [28]. A similar approach [29] was undertaken to activate the previously identified Type II restriction gene CpaAI (recognition sequence 5′-CGCG-3′) in *C. pasteurianum* [24], dispensing with the need to methylate plasmids in the *E. coli* donor using M. FnuDII methylase prior to DNA transfer.

Whilst the majority of studies have focussed on Type II systems, a recent report demonstrated that the barrier presented by Type I systems can be negated by cloning into the *E. coli* donor both the methylase and specificity subunits (HsdM and HsdS) of two type I systems (RM1 and RM2) identified in *Clostridium saccharobutylicum* NCP 262. The resultant protection of the mobilisable cloning vector in the *E. coli* Top10 donor led to the successful transfer of a shuttle vector to *C. saccharobutylicum* in a triparental mating using the conjugative donor strain CA434 [30]. These same authors went on to individually inactivate the *hsdR* components of RM1 and RM2 systems using ClosTron mutagenesis, achieving a 10-fold and 8-fold increase in transfer frequencies, respectively.

In the past, the specificity of methylation systems was determined either by showing that homologous genes encoding methylases of known specificity protected the vector to be transferred (eg. [11], [18], [21], [22], or in the case of cytosine-specific methylases, using a modification of the method of Feil et al. [31], in which all unmethylated cytosines are converted to uracil using sodium bisulphite. Subsequent sequencing of NaHSO_3_-treated DNA propagated in the target *Clostridium* reveals the identity of such cytosine residues. The development of SMRT PacBio sequencing, however, represents a step change in such determinations as it provides the opportunity to directly identify methylation patterns in genomes, both cytosine and adenosine methylation, for example in *C. pasteurianum* [32], [33] and *C. difficile* [14]. Importantly, the PACBio data obtained may be directly deposited in the REBASE PacBio database (http://rebase.neb.com/cgi-bin/pacbiolist) [34], where the necessary analysis is undertaken free of charge.

### 2.3 Establishment of gene transfer procedures

But for a brief dalliance with PEG-mediate protoplasts procedures (eg., [35]), only two methods are routinely pursued in terms of obtaining DNA transfer to clostridial hosts, electroporation and conjugative mobilisation from a shuttle host, invariably *E. coli*. In terms of conjugative plasmid transfer, the method of choice is to use conjugation between an *E. coli* donor and the clostridial recipient. The majority of methods rely on the *oriT-*mediated mobilisation of plasmids by the transfer functions of IncP plasmids, either located autonomously (as in the host CA434, which carries R702) or integrated in the genome (strain S17.1 or Sm10). The commonest origin of transfer (*oriT*) employed is that of RK2. Methods are largely based on the pioneering work of Williams et al. [36], in *C. acetobutylicum*, later replicated in *C. difficile* [17], although in some instances an alternative *oriT* region from the broad-host range transposon Tn*916,* has been used with certain strains of *C. difficile* [37]. In the case of electroporation, empirical changes are made to a multitude of parameters to achieve the highest transfer frequencies. These include preparing cells at different phases of growth, including cell weakening agents in the media, the use of different buffers for preparation of competent cells, and the different electrical parameters of the electric pulse amplified, as well as its duration (see Ref. [24] for a recent example).

### 2.4 Identification of an appropriate ‘pseudo-suicide’ replicon

The allelic exchange methodologies developed in this laboratory are all reliant on replication defective plasmids [13], [38], [39]. When such plasmids encode an antibiotic resistance gene, typically *catP,* they are maintained within the cell by antibiotic selection, thiamphenicol (Tm). Under such situations, the rate of growth of the population is determined by the rate at which the plasmid is segregated to the daughter cells. If the plasmid is endowed with a region of homology to the chromosome, then those rare cells in which the plasmids integrate via homologous recombination now have a growth advantage because every daughter cell carries a copy of the *catP* gene. The integrated sub-population, therefore, has a growth advantage over those cells in which the plasmid remains autonomous. This growth advantage manifests itself as visibly larger colonies on agar media. We have termed such plasmids, pseudo-suicide vectors [38], [40]. It follows that an early stage in the application of the roadmap to a particular clostridial species is to determine the most defective replicon of those available. This undertaking has been simplified by the creation of the pMTL80000 modular vector series [41].

The pMTL80000 vector series represent a standardised plasmid set in which each module is localised to a defined restriction fragment bounded by one of four rare 8 bp palindromic sequences, corresponding to the restriction recognition sites of the endonucleases SbfI, AscI, FseI and PmeI [41]. Modules correspond to the Gram-negative replication region (for maintenance in *E. coli*), the Gram-positive replicon (for maintenance in clostridia), an antibiotic resistance gene (for selection in both *E. coli* and *Clostridium*), and an application specific module (eg., reporter gene, promoter, multiple cloning sites). These modules are always arranged in the same order, *viz.,* PmeI-SbfI (Gram-negative replicon), SbfI-AscI (application specific module), AscI-FseI (Gram-positive replicon) and FseI-PmeI (antibiotic resistance gene). At the time, 18 modules were available, including 5 different Gram-positive replicons, those of the plasmids pBP1 (*C. botulinum*), pCB102 (*Clostridium butyricum*), pCD6 (*C. difficile*) and pIM13 (*Bacillus*). The modules are numbered to allow the easy identification of the components present in any particular plasmid. This system allows the combinatorial construction of shuttle plasmids from modules in the standard format. It also provides for the quick and easy modification of existing pMTL80000-based plasmids. All of the pMTL allelic exchange vectors, transposon vectors and ACE vectors described in this review conform to this modular format. Moreover, numerous other modules have been added to the system, including new replicons. Updates can be found at http://chainbiotech.com/modular-plasmids/.

**Fig. 1:**
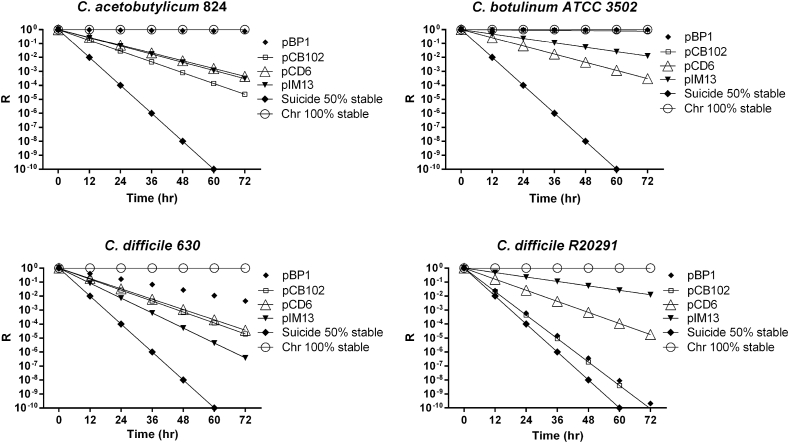
Relative stability of the various modular replicons in different species and strains of *Clostridium*. R = Plasmid loss in media lacking supplementation with antibiotic as a function of time and measured as loss of antibiotic resistance. Each replicon based plasmid is compared to the stability of a cell carrying the antibiotic gene in the chromosome. For comparison, the projected rate of loss of a population carrying a plasmid that cannot replicate (‘Suicide’) is given.

To determine the most defective replicon, the vectors are transferred to the target clostridial strain and their segregational stability assessed. This can be determined either by measuring the growth rate in the presence of antibiotic, or by growing in the absence of antibiotic and then estimating the number of cells that have lost the plasmid. The latter involves either plating cells on agar media with and without antibiotic and comparing the cfu/ml, or by plating onto media lacking antibiotics and then patch plating onto agar media with and without antibiotic (Fig. 1). It should be noted that aside from the different properties of replicons in the different clostridial species, they can also show wide variation in different strains of the same species. For example, plasmids based on the pBP1 replicon are the least stable in the *C. difficile* strain R20291, whereas this represents the most stable replicon in strain 630 (see Fig. 1).

### 2.5 Generation of a ClosTron pyrE mutant to formulate FOA-selective media

The *pyrE* gene encodes orotate phosphoribosyl transferase, responsible for the conversion of the pyrimidine intermediate orotic acid into orotidine 5′-monophosphate (OMP). FOA is an analogue of orotic acid and is converted by the same enzyme into 5-fluoroorotidine monophosphate (5-FOMP) which is subsequently converted to 5-fluorouridine monophosphate (5-FUMP), instead of UMP. Accumulation of 5-FUMP is toxic and leads to cell death [42]. It follows that the inactivation of *pyrE* prevents the accumulation of 5-FUMP and therefore, confers on the host a FOA^R^ phenotype.

The first step of the roadmap is to generate a *pyrE* mutant using ACE. The isolation of this mutant is facilitated by the fact that such mutants become resistant to FOA. As it is not clear what FOA concentration to use in selective media it is preferable to use ClosTron technology to rapidly generate a *pyrE* mutant, and then use that mutant to establish the FOA supplemented media required to distinguish *pyrE* mutants from wild type. The ClosTron is one of the most used clostridial gene knock-out systems and is a derivative of the Sigma Aldrich Targetron system [43], [44], [45]. By making a handful of nucleotide changes to the group II intron encoding region, the intron can be directed to insert into almost any region within the genome. Through the use of a Retrotransposition-Activated Marker (RAM) based on the *ermB* gene, successful insertion is selected on the basis of acquisition of resistance to erythromycin. The re-targeted region is designed using an online re-targeting algorithm (www.clostron.com), and an order placed with DNA2.0 for both the synthesis of the retargeted region AND its custom cloning into the ClosTron vector. Re-targeted ClosTrons are delivered ready for use in 10–14 days, allowing mutants to be isolated 5–7 days after receipt. This dispenses with the need to purchase Sigma Aldrich kits, or pay to use their algorithm.

Standard protocols [44], [45], [46] are deployed to implement ClosTron technology in the chosen *Clostridium*, to generate a *pyrE* (encoding orotate phosphoribosyl transferase) mutant, requiring exogenous uracil. Once obtained, the concentration of FOA required to select for *pyrE* minus cells is determined empirically. As *pyrE* mutants are auxotrophic, the media also has to contain exogenous uracil. Generally speaking, the level of exogenous uracil added is in the range of 5–50 μg/ml, while the FOA supplementation can be as low as 800 μg/ml (*C.beijerinckii*) to as high as 3.5 mg/ml (*Clostridium sporogenes*).

### 2.6 The use of ACE and the FOA-selective media to create a pyrE deletion mutant

Having established the most defective Gram-positive replicon, an ACE (Allele-Coupled Exchange) [47] vector is constructed using this replication region based on the pMTL80000 modular format [41] to inactivate *pyrE.* Following integration of the ‘pseudo-suicide’ plasmid by single-crossover recombination, the system is designed such that during the desired second recombination event, a plasmid borne allele becomes ‘coupled’ to a genome located allele which leads to the creation of a new selectable (in this case FOA^R^) allele, allowing the isolation of double-crossover cells. The use of highly asymmetric homology arms dictates the order of recombination events. A long, right homology arm (RHA) directs the first recombination event (plasmid integration) and a much shorter left homology arm (LHA) directs the second recombination event (plasmid excision). The ease and rapidity of ACE allows the sequential extension of operon size and complexity through repeated cycles of the method, as demonstrated through iterative insertion of the entire lambda genome (48.5 kb) into the *C. acetobutylicum* genome [47] as wells as synthetic mini-cellulosome operons [48].

As indicated (section 2.7), following transfer of the ACE vector into the cell, single cross-over integrants are selected based on faster growing, larger colonies. Such integrants are invariably integrated via the LHA, due to its greater size compare to the RHA [47]. These faster growing colonies are then streaked out onto minimal media lacking thiamphenicol and supplemented with FOA and uracil at the concentration determined (section 2.7) using the ClosTron mutant. Those FOA^R^ cells that arise will represent deletion mutants in which the desired second crossover event has occurred and the excised plasmid has been lost. Phenotypically they are, therefore, FOA^R^, uracil minus and Tm sensitive (^S^). Their authenticity is checked by using oligonucleotide primers that flank the *pyrE* gene to PCR amplify a DNA fragment, which in addition to being of a smaller size compared to the wild type, is confirmed to encompass the expected deletion event by nucleotide sequencing.

### 2.7 Implementation of allelic exchange methodologies

As the *pyrE* mutant is now resistant to FOA, the introduction of a functional copy of a *pyrE* gene will lead to restoration of sensitivity. As such, the introduced gene can be used as a counter selection marker. Accordingly, KO pseudo-suicide vectors can be constructed carrying a suitable KO cassette (composed of equal sized homology arms flanking the gene to be inactivated), an antibiotic resistance marker (*catP*), a heterologous (to avoid unwanted homologous recombination) functional *pyrE* gene and the selected (section 2.4) defective replicon. The plasmid is introduced into the cell and clones in which the plasmid has integrated via homologous recombination, between one or other of the two homology arms and the corresponding complementary DNA in the chromosome, selected on the basis of their larger colony size on agar media supplemented with Tm. Selection and restreaking of the faster growing colonies allows the isolation of single crossover integrants, as determined by PCR screening using an appropriate combination of primers complementary to regions flanking the homology arms and vector encoded sequences. The isolation of pure single crossover populations is essential as the presence of substantive sub-populations of cells carrying autonomous plasmids can lead to high counts of spurious mutants in the presence of the counter selection agent.

Examples of the KO vectors constructed based on this principle are pMTL-YN3 and pMTL-YN4, which are used for KO in *C. difficile* strain 630 and the PCR-ribotype 027 strain R20291, respectively [39]. The former vector uses the pCB102 replicon [49], due to its comparatively greater defectiveness in strain 630, while plasmid pMTL-YN4 is based on the pBP1 replicon which is the most unstable replicon in strain R20191. In the case of *C. acetobutylicum* ATCC 824, the replicon of choice and the one used in the KO vector pMTL-ME3 [13] is that of pIM13. The heterologous *pyrE* gene used in the case of both organisms was that of *C. sporogenes* ATCC 15579. Traditionally KO cassettes may be individually generated (PCR) as separate Left and Right Homology Arms (LHA and RHA) that are sequentially cloned using created (part of the primer) restrictions sites into corresponding vector restriction sites. Alternatively, they can be commercially synthesized, as either the entire DNA fragment or, if appropriately designed, they can be assembled from smaller fragments using procedures such as G-Blocks [50], USER cloning [51], ligase cycling [52] or Golden Gate [53]. Alternatively, the two DNA fragments comprising the LHA and RHA may be joined prior to cloning using Splicing Overlap Extension PCR (SOE) [54].

The robustness and reliability of the method was initially demonstrated in *C. difficile* through the creation of in-frame deletions in *spo0A*, *cwp84*, and *mtlD* in strain 630Δ*erm* and *spo0A* and *cwp84* in R20291 [39], and in *C. acetobutylicum* ATCC 824 the *spo0A, cac824I*, *amyP* and *glgA* genes [13]. The procedure has proven equally effective using the alternative negative selection marker *codA* in both *C. difficile* [39] and *C. acetobutylicum* [13]. This gene encodes cytosine deaminase, (EC 3.5.4.1) which catalyzes the conversion of cytosine to uracil. It also converts the innocuous pyrimidine analogue 5-fluorocytosine (FC) into the highly toxic 5-fluorouracil (FU). FU toxicity occurs as a result of the irreversible inhibition of thymidylate synthase, a key enzyme in nucleotide biosynthesis.

### 2.8 ACE correction vectors

**Fig. 2:**
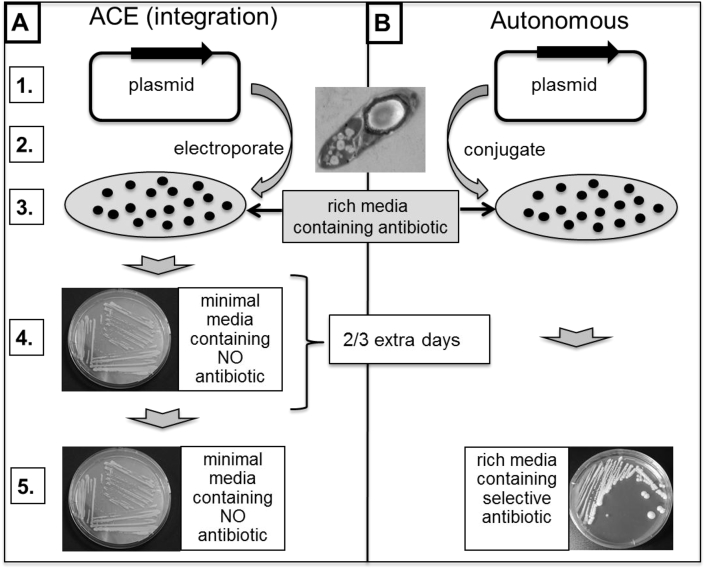
**Comparison of the steps required for complementation using ACE integration [A] or autonomous [B] vectors**. Step are: (1) Plasmid construction; (2) Transfer to clostridia (electroporation or conjugation); (3) development of transformant/transconjugant colonies on rich agar containing antibiotic relevant to the resistance gene present on the plasmid backbone; (4) in the case of [A] only, restreaked on minimal media lacking uracil (only double crossover integrants grow) and antibiotic supplementation, and; (5) purification of complemented clonal populations, in the case of [A] on rich media containing antibiotic to select for the plasmid, and in the case of [B] rich media containing antibiotic. ACE integration [A] is characterised by being at appropriate gene dosage, requires no supplementing antibiotic as the complementing gene is entirely stable, this eliminates antibiotic effects on phenotype and, therefore dispenses with the requirement for vector only control. The use of autonomous vectors [B] results in high gene dosage that can affect phenotype, requires antibiotic supplementation to maintain the plasmid which can affect phenotype and necessitates the inclusion of an additional vector only control.

Once the *pyrE* mutant is generated, an ACE correction vector is constructed, designed to restore *pyrE* to wild type. In this instance, following the isolation of a single crossover integrant, the desired double crossover event is simply selected by plating on media lacking uracil. That is to say, the auxotrophic mutant is converted back to prototrophy. The nature of the deletion is such that reversion to prototrophy cannot occur by any other means than ACE-mediated replacement of the defective allele with a wild type version. In other words, there are no false positives. The prototrophic cells now become FOA sensitive (^S^). Crucially, the system provides the in parallel opportunity to complement the mutant at an appropriate gene dosage, through the insertion of a functional wild type copy of the gene, into the genome, either under the control of its native promoter or the strong P_fdx_ promoter (derived from the ferredoxin gene of *Clostridium sporogenes*), concomitant with restoration of the *pyrE* allele back to wild type [13], [39]. The extra effort involved in the deployment of ACE vectors compared to the use of autonomous complementation vectors is minimal (Fig. 2). They require the same amount of effort in terms of construction and transfer into the desired bacterial host. Mutants transformed with autonomous complementation plasmids need to be purified by restreaking, whereas an ACE complementation transformant merely needs to be restreaked onto minimal agar media lacking uracil, and those colonies that grow purified by restreaking. The extra effort, therefore, equates to the time it takes for uracil prototrophic colonies to develop, ca. 2–3 days in the case of *C. difficile* for instance (Fig. 2). The efficiency of ACE is such that success is assured and moreover, false positives cannot arise as reversion of the *pyrE* deletion is impossible. Although the effort required for ACE-mediated complementation is minimal, the benefits are considerable. It avoids the phenotypic effects frequently observed with high copy number plasmids and dispenses with the need to add antibiotic to ensure the retention of the complementing plasmid. Such antibiotic addition can affect phenotype [see 39] and necessitate the inclusion in any phenotypic assessments of the mutant a vector only control. Moreover, the *pyrE* allele represents an ideal position where other application specific modules may be inserted into clostridial genomes, such as a sigma factor to allow deployment of a *mariner* transposon [55], hydrolases for degrading complex carbohydrates [48], therapeutic genes in cancer delivery vehicles [56] or the addition of an *ermB* gene to improve the reproducibility of the virulence of the NAP1/B1/027 epidemic strain R20291 in the hamster model of infection [57].

The benefits of the presence of the *pyrE* locus are such, that there is a rational argument for using *pyrE* mutant hosts, and their cognate ACE correction vectors with any particular mutagen, including the ClosTron and any of the alternative negative selection markers developed in recent years. These include the *E. coli* genes *codA* (cytosine deaminase) and *mazF* (mRNA interferase) exemplified in *C. difficile* [38] and *C. acetobutylicum* [13], [58], respectively, and the *Thermoanaerobacterium saccharolyticum tdk* (thymidylate synthetase) and *Clostridium thermocellum hpt* (hypoxanthine phosphoribosyl transferase) genes, which were used to make knock-outs in *C. thermocellum* [59]. The use of *pyrE* host would also find utility in the recently published recombineering approach developed for use in *C. acetobutylicum* and *C. beijerinckii* [60], as well as allelic exchange mutants made using CRISPR genome editing [61], [62], [63].

**Fig. 3:**
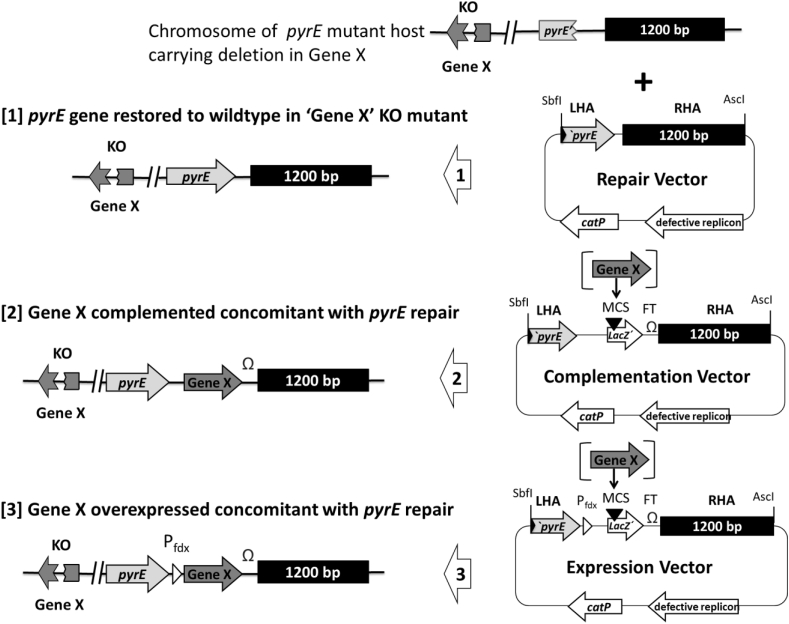
**The three types of ACE integration vectors used to restore the *pyrE* allele to wildtype in the *pyrE* mutant host in which a mutation (Gene X) has been made by allelic exchange**. Integration cassettes are modular and inserted between the *Sbf*I and *Asc*I sites of the pMTL80000 vectors. Each vector has a long (1200 bp) Right hand homology arm (RHA) and a shorter (300 bp) left homology arm (LHA). The latter is composed of the 3′-end of the *pyrE* gene, while former comprises the 1200 bp region of the chromosome from immediately downstream of the *pyrE* gene. In the case of the ACE Correction vector [1], the 300 bp and 1200 bp regions are in effect a continuous 1500 bp region of homology to the host chromosome in this region. In the case of the Complementation [2] and Overexpression [3] vectors, the two homology arms are separated by a region of DNA comprising a *lacZ* containing multiple cloning site (MCS) region and a downstream transcriptional terminator (FT). The Expression vector additionally contains a strong promoter (P_*fdx*_) from the *C. sporogenes* ferredoxin gene immediately before the *lacZ′* gene. Using the MCS, the complementation and expression plasmid variants allow delivery of a functional copy of a knocked-out gene (Gene X) under the control of its native promoter, for complementation studies, or under the control of the strong promoter of the *C. spororgenes* ferredoxin gene (P_*fdx*_), to allow an assessment of the effect of overexpressing the gene. In every case, integration if the ACE plasmid is initially via the longer RHA. Subsequent plasmid excision via the LHA restores the *pyrE* allele to wildtype, allowing the former *pyrE* minus host to grow on minimal media lacking uracil.

A suite of *pyrE* ACE vectors are available (Fig. 3) to either: correct the mutant *pyrE* allele of deletion mutants made in either *C. difficile* or *C. acetobutylicum.* In *C. difficile* strains the correction vectors for strains 630Δ*erm*Δ*pyrE* and R20291Δ*pyrE* are pMTL-YN1 and pMTL-YN2, respectively. Those vectors that allow the simultaneous complementation of an inactivated gene concomitant with restoration of prototrophy are pMTL-YN1C and pMTL-YN2C, while those that bring about overexpression of the complementing gene are pMTL-YN1X and pMTL-YN2X. In *C. acetobutylicum* the respective vectors are pMTL-ME6 (correction vector), pMTL-ME6C (complementation vector) and pMTLME6X (overexpression vector). Equivalent vector sets, are available in this laboratory for *C. beijerinckii*, *C. botulinum*, *C. perfringens, C. sporogenes, C. pasteurianum*, *Clostridium ljungdahlii* and *C. autoethanogenum*. Further vectors are also under development for a number of other clostridial species.

### 2.9 Creation of random mutant libraries through the mariner transposon mutagenesis

One exemplification of the utility of ACE and the *pyrE* locus for inserting application specific modules is that used to derive a universal transposon system for *Clostridium* sp [55]. In early work, we had demonstrated the utility of a *mariner* based plasmid system (pMTL-SC1) in *C. difficile* [40] in which the *mariner* transposase gene was placed under the control of the promoter (P_tcdB_) of the *C. difficile* toxin B gene, *tcdB.* This promoter (P_tcdB_) is exclusively recognised by a specialised class of sigma factor, TcdR, which belongs to a family that is unique to a handful of toxinogenic clostridial species [64]. As *E. coli* does not produce an analogous sigma factor, the promoter is poorly recognised by this host, a feature that prevents transposon activity in *E. coli* prior to transfer of the vector. Avoidance of transposition activity in the donor strain prior to transfer to the clostridial recipient is a desirable attribute as transferred plasmids could potentially either become devoid of the *catP*-based min-transposon or indeed in some way functionally affected by its insertion into the vector at a new position. Essentially, the system may be considered as a conditional expression system, where transposase expression is limited to the clostridial host.

As other clostridial species lack a functional equivalent to TcdR we reasoned that if we were to introduce the encoding gene into genome at the *pyrE* locus using ACE then the pMTL-SC1 transposon vector should be functional in the new host. Accordingly, the *tcdR* gene was inserted into the genomes of both *C. acetobutylicum* and *C. sporogenes* using the ACE complementation vector pMTL-ME6C (section 2.7) and a functionally equivalent plasmid developed for *C. sporogenes* [55]. Successful integrants were selected merely on the basis of restoration of uracil prototrophy (see section 2.7). In both cases, the genes were cloned without the *tcdR* promoter region and were therefore reliant on the upstream promoters responsible for *pyrE* expression. This level of expression apparently had no effect on the phenotype of the strains generated based on the observed absence of any effects on growth rate, sporulation frequency and measured metabolic products [55]. The level of expression was, however, sufficient for effective expression of *tcdR*, as transposition of the min-transposon was readily detected at a frequency of 2.6 (±0.6) × 10^−4^ and 3.2 (±0.5) × 10^−4^ in *C. acetobutylicum* and *C. sporogenes,* respectively [55]. As was previously the case with *C. difficile,* inverse PCR on the *C. acetobutylicum* and *C. sporogenes* transposon mutants demonstrated that just a single insertion had taken place in the overwhelming majority of cases (98.3% and 96.7%, respectively) and that insertion had taken place principally within protein coding sequences (respectively, 83.6% and 79%). The latter frequency is consistent with the fact that 80% of the clostridial genome is protein coding. The utility of the system was further exemplified by the isolation of mutants phenotypically affected in sporulation/germination as well as autotrophic strains which could no longer grow on minimal media [55].

The utility of the system was further improved by the development and deployment of a plasmid delivery vehicle that was conditional for replication. This was achieved by placing an IPTG inducible promoter upstream of the pCB102 replicon. In the absence of inducer the plasmid replicated normally. Upon addition of IPTG, the plasmid (pMTL-YZ14) was rapidly lost: 80% in the case of *C. sporogenes* and 100% in the case of *C. acetobutylicum* [55]. The ability to rapidly lose the plasmid represented a considerable improvement on pMTL-SC1, which was based on a pseudo-suicide replicon and required a minimum of two passages of the recipient bacteria to eradicate the *Himar1 C9* transposase encoding plasmid. The conditional vector has been shown to function effectively in *C. beijerinckii*, *C. botulinum* and *C. autoethanogenum* but cannot be used in *C. difficile* (unpublished data). The latter observation is because *C. difficile* apparently does not take up IPTG. Functionality in this particular clostridial host, or indeed any clostridial host where a similar obstacle is encountered, will require the substitution of the IPTG inducible promoter with a different inducible system, eg., the anhydrotetracycline inducible system exemplified in *C. difficile* [65] or the lactose inducible system of *C. perfringens* [66].

## 3 Roadmap outcome

**Fig. 4:**
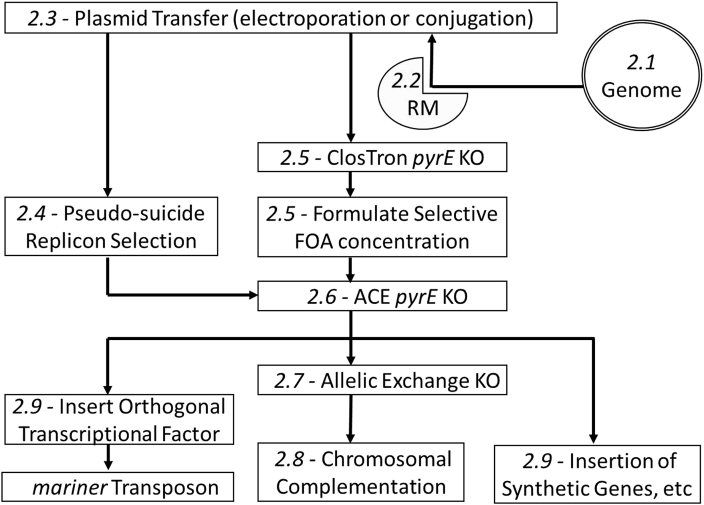
Schematic representation of Roadmap stages. For details refer to relevant section (2.1 to 2.9) in the text.

The key steps involved in the roadmap are summarised in Fig. 4. Through implementation of the outlined procedures it is possible to formulate a generally applicable toolbox for use in potentially any *Clostridium* spp. To date, provided DNA transfer is obtained, we have not encountered any clostridial species where this technology cannot be applied. Thus, all clostridia contain the requisite pyrimidine pathway, the inactivation of which leads to uracil auxotrophy and FOA^R^. ClosTron technology appears universally applicable and at least one, most usually all, of the modular replicons available have proven functional. In the unlikely event that they are not, the modular nature of our vectors mean that a new functional replicon can be rapidly substituted. Aside from the use of *pyrE* alleles as counter selection markers, their most useful attribute resides in the use of the mutant allele, in combination with ACE, as a locus for genome insertion, be it for complementation studies or for the insertion of application specific modules. The case for using such hosts for all mutational studies, regardless of the mutagen, is compelling.
